# Reporting items for systematic reviews and meta-analyses of acupuncture: the PRISMA for acupuncture checklist

**DOI:** 10.1186/s12906-019-2624-3

**Published:** 2019-08-12

**Authors:** Xiaoqin Wang, Yaolong Chen, Yali Liu, Liang Yao, Janne Estill, Zhaoxiang Bian, Taixiang Wu, Hongcai Shang, Myeong Soo Lee, Dang Wei, Jinhui Tian, Bin Ma, Yongfeng Wang, Guihua Tian, Kehu Yang

**Affiliations:** 1 Evidence-Based Medicine Center, School of Basic Medical Sciences, Lanzhou University, Lanzhou, China; 2 Evidence-Based Social Science Research Center, School of Public Health, Lanzhou University, Lanzhou, China; 3 Key Laboratory of Evidence Based Medicine and Knowledge Translation of Gansu Province, Lanzhou, China; 4 WHO Collaborating Centre for Guideline Implementation and Knowledge Translation, Lanzhou, China; 5 Clinical Division, School of Chinese Medicine, Hong Kong Baptist University, Kowloon Tong, Hong Kong; 6 Institute of Global Health, University of Geneva, Geneva, Switzerland; 7 Institute of Mathematical Statistics and Actuarial Science, University of Genewa, Geneva, Switzerland; 8 Chinese Cochrane Centre, Chinese Evidence-Based Medicine Centre, West China Hospital, Sichuan University, Chengdu, China; 9 Dongzhimen Hospital of Beijing University of Chinese Medicine, Beijing, China; 10 Korea Institute of Oriental Medicine, Daejeon, South Korea; 11 Department of Public Health Sciences, Karolinska Institute, Stockholm, Sweden; 12 Gansu University of Chinese Medicine, Lanzhou, China

**Keywords:** PRISMA, Acupuncture intervention, Systematic review

## Abstract

**Background:**

Acupuncture is widely used worldwide, and systematic reviews on acupuncture are increasingly being published. Although acupuncture systematic reviews share several essential elements with other systematic reviews, some essential information for the application of acupuncture is not covered by the Preferred Reporting Items for Systematic reviews and Meta-Analyses (PRISMA) statement. Considering this, we aimed to develop an extension of the PRISMA statement for acupuncture systematic reviews.

**Methods:**

We used the PRISMA statement as a starting point, and conducted this study referring to the development strategy recommended by the EQUATOR network. The initial items were collected through a wide survey among evidence users and a review of relevant studies. We conducted a three-round Delphi survey and one-day face-to-face meeting to select items and formulate the checklist. After the consensus meeting, we drafted the manuscript (including the checklist) and sent it to our advisory experts for comments, following which the checklist was refined and circulated to a group of acupuncture systematic review authors for pilot test. We also selected a sample of acupuncture systematic reviews published in 2017 to test the checklist.

**Results:**

A checklist of five new sub-items (including sub items) and six modified items was formulated, involving content related to title, rationale, eligibility criteria, literature search, data extraction, and study characteristics. We clarified the rationales of the items and provided examples for each item for additional guidance.

**Conclusion:**

The PRISMA for Acupuncture checklist is developed for improving the reporting of systematic reviews of acupuncture interventions.

**Trial registration:**

We have registered the study on the EQUATOR network (http://www.equator-network.org/library/reporting-guidelines-under-development/#91).

**Electronic supplementary material:**

The online version of this article (10.1186/s12906-019-2624-3) contains supplementary material, which is available to authorized users.

## Background

Having been applied for thousands of years for prevention, treatment and rehabilitation for various diseases, acupuncture is currently used in more than 140 countries worldwide [1–3]. With a large number of randomized controlled trials and systematic reviews of acupuncture published every year, the importance to improve reporting quality of acupuncture studies has been highlighted by stakeholders including both researchers and users of acupuncture evidence. The reporting quality of clinical trials of acupuncture has improved after the implementation of STandards for Reporting Interventions in Clinical Trials of Acupuncture (STRICTA) [4, 5], but no reporting standards for acupuncture systematic reviews have been established yet.

The Preferred Reporting Items for Systematic reviews and Meta-Analyses (PRISMA) has been developed and is used by academic institutions and journals worldwide to promote the reporting quality of systematic reviews and meta-analyses [6, 7]. Extensions of PRISMA, such as PRISMA for Harms (for reviews including harm outcomes), Protocols, Equity, Abstracts, Individual Patient Data, and Network meta-analyses [8–12], are continuously being developed and published for reporting important aspects in these reviews. Systematic reviews of acupuncture share some essential elements with reviews of other topics. Some areas, such as the types of acupuncture, the selection of acupoints, depth and duration, are however not covered by PRISMA, although they are vital for evidence users to understand the intervention process, for guideline developers to formulate practical recommendations, and for clinical professionals to implement acupuncture in practice [13–16]. We therefore aimed to develop a reporting checklist for acupuncture systematic reviews and meta-analyses (PRISMA for Acupuncture) based on the PRISMA statement.

## Aim and scope

The aim of the PRISMA for Acupuncture checklist is to optimize reporting of systematic reviews focusing on acupuncture interventions for specific conditions. The main group of target users of PRISMA for Acupuncture are authors of systematic reviews on acupuncture, journal editors, peer reviewers and methodologists. We hope this checklist is a useful and practical tool for these audiences and will improve reporting and usability of systematic reviews on acupuncture.

## Methods

**Fig. 1:**
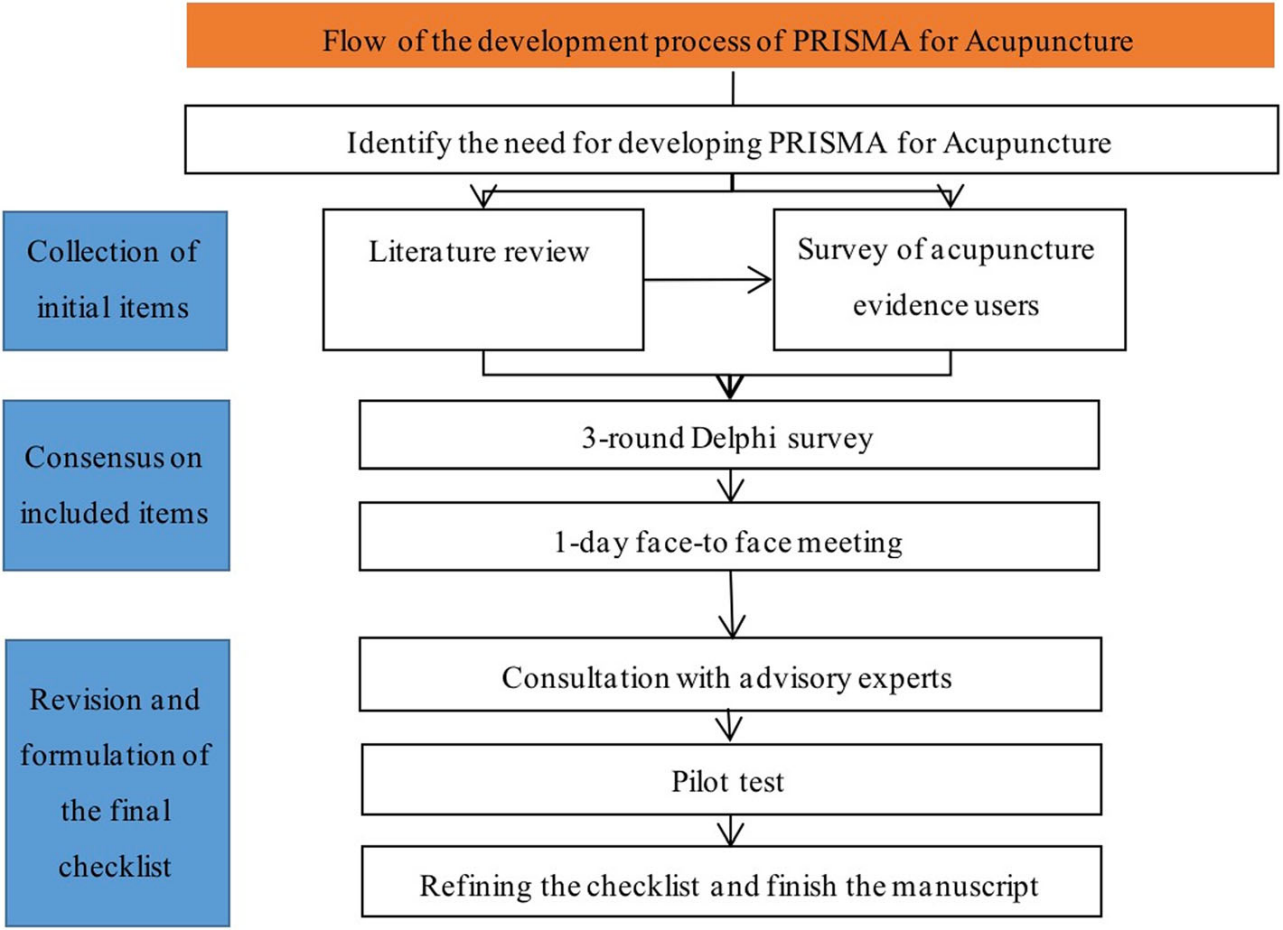
The development process of the PRISMA for Acupuncture checklist

The PRISMA for Acupuncture working group registered the project on the Enhancing the QUAlity and Transparency Of health Research (EQUATOR) Network [17]. We used the PRISMA statement as a starting point, and conducted this study referring to the EQUATOR guidance for developing health research reporting guidelines [18]. The selection of items consisted of three steps: 1) collecting and framing the initial items, 2) scoring and selecting the items by experts through Delphi consensus; and 3) discussing and approving the checklist in a face-to-face meeting. The advisory experts provided comments to revise the checklist and manuscript, and pilot tests were applied to seek feedback to refine the final checklist. (Fig. 1).

### Selection of the initial items

After establishing the PRISMA for Acupuncture working group, we conducted a review of systematic reviews for acupuncture [19] and a survey for end users of acupuncture evidence [16] to identify the initial set of items for consideration. For the survey, we collected the ideas of respondents about importance of the proposed eight items from the review, after which they could also suggest further items. In order to improve the representativeness of this survey, we conducted a multi-center questionnaire survey in five cities (Beijing, Shanghai, Nanjing, Chengdu, and Lanzhou) located in different parts of China. To ensure sufficient number of responses, two members of the working group visited these places in person between April and June 2014, where they distributed and collected the questionnaires, without influence the response process. Respondents included acupuncture practitioners, researchers and methodologists working on acupuncture research, and postgraduates with more than 1 year of experience of practicing acupuncture. A total of 269 stakeholders participated in this survey. The survey received ethical approval from the Research Ethics Committee of the first hospital of Lanzhou University, Lanzhou, China. Details of the survey and results have been published before [16].

### Delphi consensus process

Based on the results from the first step, we selected the potential items for reporting in acupuncture systematic reviews. The initial items focused mainly on details of the acupuncture intervention. We also considered items relevant to other aspects of systematic reviews on acupuncture, such as sources for literature search and selection of studies. We then assembled a multidisciplinary group of experts based on their specialties and experience, and balanced the numbers of clinicians, researchers and healthcare providers when forming the panel. We invited 32 experts, of whom 29 agreed to participate, including senior acupuncture practitioners, methodologists of reporting guidelines and systematic reviews, epidemiologists, journal editors and statisticians.

We conducted a three-round Delphi process from October 2015 to July 2016, aiming to achieve consensus on essential items that should be included in the PRISMA for Acupuncture checklist and to identify items that required discussion at the face-to-face meeting. All 29 participants responded during the three rounds of the Delphi survey. In the Delphi survey, participants were asked to score each item using a 5-point Likert scale ranging from “of no importance” to “very important” [20, 21]. In the first round, the Delphi experts were invited to score all initially included items as well as suggest any additional potentially relevant items. The second round included any items that did not reach consensus and any new items from the first round. The third round involved items that did not reach consensus during the previous rounds. We conducted the Delphi surveys through emailing every expert separately and sent the summary of the previous round of survey in every next round, without mentioning any identical information of the other experts.

After each round, the score was calculated with the below formula, where *N*_*i*_ represents the number of respondents who gave the score*.* We consulted with the leading authors as well as the statistician in the Reporting Items for practice Guidelines in HealThcare (RIGHT) working group and used the same scale and formula from the RIGHT statement, where both the consensus level and the weight of responses were considered. *N*_*i*_ means the number of experts who chose specific “*i”* in the Likert scale (1 to 5), and items with a score greater than or equal to 75% were included [22]. An anonymized summary of the results of each round was sent to all participants through email. The survey was sent and collected through one specific email account, and one member of the working group (XW) monitored this email account.

$$ 100\%\ast \left({N}_5+0.75\ast {N}_4+0.5\ast {N}_3+0.25\ast {N}_2\right)/\left({N}_5+{N}_4+{N}_3+{N}_2+{N}_1\right) $$

### Face-to-face meeting

After the Delphi process, we created a draft checklist with the included items. Ten experts, including acupuncture practitioners, methodologists, reporting guideline developers, journal editors and a statistician, attended a one-day face-to-face meeting in Lanzhou, China, on 12th October 2016. During the meeting, the results of the Delphi process were presented, followed by a discussion and refinement of each item. The participants then voted about the inclusion of each proposed item and decided the precise wording. We present only the aggregated results to maintain the anonymity of the participants. At the end of the meeting, the ten experts reviewed the checklist again to confirm that their comments were appropriately understood and considered.

The checklist was then developed in accordance with the EQUATOR template and presented in line with the PRISMA checklist.

### Consultation with advisors

After the face-to-face meeting, we circulated the manuscript to advisory experts for additional comments. During consultations, the wording and presentation of the checklist and manuscript were further revised. Following this, the checklist was pilot tested.

### Pilot tests

To identify practical challenges with any of the items, members of the PRISMA for Acupuncture working group applied the checklist to investigate the reporting condition of a sample of acupuncture systematic reviews published in 2017. In addition, we also conducted an online survey to corresponding authors of systematic reviews on acupuncture to obtain further comments on the utility and clarity of the checklist. Feedback from all pilot tests were used to refine the wording and presentation of the final checklist.

## Results

### Delphi process

**Fig. 2:**
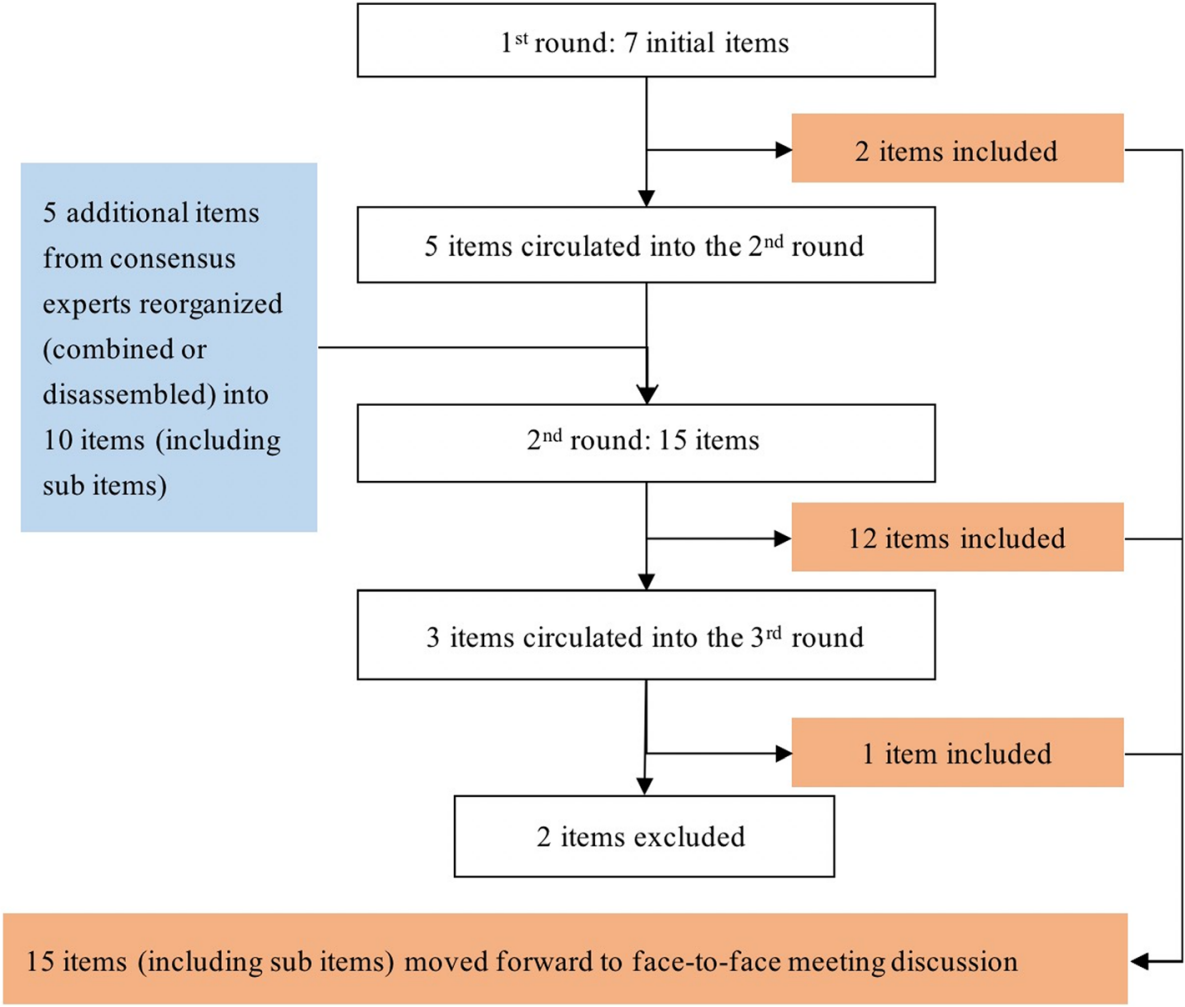
The result of the Delphi process

Based on the results of the collection of initial items, we included seven items for the first round of the Delphi process. The Delphi results were as follows (Fig. 2):

1. Two items that scored greater than 75% were included after the first round. The experts gave their feedback for revising the remaining items. No additional items were suggested by the panel.
2. For the remaining items, we combined or split some items into sub-items, and revised the wording according to feedback from the first round. A total of 15 items were surveyed in the second round, and the results showed a high consensus on twelve items with no more major changes. While the remaining three items were modified according to comments and entered into the third round.
3. By analyzing the feedback from the third round of the Delphi process, one item was added from the third round of the Delphi process.

The results of the 3-round Delphi process are shown in Additional file 1.

### Consensus meeting, consultation with advisors and the pilot test

Experts attending the face-to-face meeting discussed each item to improve their practicability and accuracy and approved all the 15 items included in the Delphi stage. The two items excluded in the Delphi survey were confirmed for removal.

The advisory experts reviewed the checklist and manuscript, provided comments about the wording of items, as well as if the item should be considered as new, or as an elaboration of an existing PRISMA item to enhance the relevance, as done in other extensions of PRISMA [23]. At the same time, we simplified some items about the description of acupuncture interventions by referencing to the Template for Intervention Description and Replication (TIDieR) checklist and guide, instead of clarifying the details in sub-items.

We then formulated a checklist for a pilot test, where a sample of 27 systematic reviews on acupuncture published in 2017 were assessed. Thirteen out of 56 authors (23%) of acupuncture systematic reviews from the US, Australia, Taiwan, and Mainland China replied and commented on the checklist (Additional file 2). After integrating the feedback from the pilot test, one item about De-qi under the **Data abstraction** domain was moved to the **Study characteristics** domain, and several minor modifications were made to improve the wording of the items. Finally, a checklist of five new items (marked with‡) and six modified items (marked with^*^) was formulated (Table 1). The rationale of each item is presented below. The serial numbers correspond to the items of the original PRISMA statement.

**Table 1:** PRISMA for acupuncture checklist

Subjects	PRISMA for Acupuncture
Title	
Title	1^*^ Identify the report as a systematic review, meta-analysis, or both; if applicable, state the specific type of acupuncture treatment, such as manual acupuncture or electroacupuncture.
Abstract	
Structured summary	2^†^ Provide a structured summary including, as applicable: background; objectives; data sources; study eligibility criteria, participants, and interventions; study appraisal and synthesis methods; results limitations; conclusions and implications of key findings; systematic review registration number.
Introduction	
Rationale	3^*^ Describe the rationale for what is already known about acupuncture for the target condition in the background; if applicable, state what is already known about the specific types of acupuncture to be studied, and describe whether there is any difference of the effects among different types of acupuncture.
Objectives	4^†^ Provide an explicit statement of questions being addressed with reference to participants, interventions, comparisons, outcomes, and study design (PICOS)
Methods	
Protocol and registration	5^†^ Indicate if a review protocol exists, if and where it can be accessed (e.g., web address), and, if available, provide registration information including registration number.
Eligibility criteria	6^†^ Specify study characteristics (e.g., PICOS, length of follow-up) and report characteristics (e.g., years considered, language, publication status) used as criteria for eligibility, giving rationale. 6a.1^‡^ Describe the diagnostic criteria of the target condition in Western medicine. 6a.2^‡^ If applicable, describe the diagnostic criteria in terms of Traditional Medicine, such as Traditional Chinese Medicine. 6b^‡^ Describe the types of acupuncture to be included, such as traditional acupuncture, electroacupuncture, or fire acupuncture. 6c^‡^ If applicable, report measures for therapeutic effects using the terminology of either traditional medicine (e.g. syndrome score for syndrome remission) or Western medicine (e.g. pain intensity).
Information sources	7^*^ Describe all sources of information (e.g., databases with dates of coverage, contact with study authors to identify additional studies) in the search, and report the date of the last search. If applicable, report the databases or complementary search methods for acupuncture or traditional medicine.
Search	8^*^ Present full electronic search strategy for at least one commonly used database (e.g. MEDLINE), including any limits used, such that it could be repeated. If applicable, include the full search strategy for at least a Western and a traditional medicine database for each systematic review where both were used.
Study selection	9^†^ State the process for selecting studies (i.e., screening, eligibility, included in systematic review, and, if applicable, included in the meta-analysis).
Data collection process	10^†^ Describe method of data extraction from reports (e.g., piloted forms, independently, in duplicate) and any processes for obtaining and confirming data from investigators.
Data items	11^*^ List and define all variables for which data were sought (e.g., PICOS, funding sources) and any assumptions and simplifications made; describe data items about details of acupuncture interventions and controls (e.g., sham acupuncture) referring to TIDieR when applicable.
Risk of bias in individual studies	12^†^ Describe methods used for assessing risk of bias of individual studies (including specification of whether this was done at the study or outcome level), and how this information is to be used in any data synthesis.
Summary measures	13^†^ State the principal summary measures (e.g., risk ratio, difference in means).
Synthesis of results	14^†^ Describe the methods of handling data and combining results of studies, if done, including measures of consistency (e.g., I^2^) for each meta-analysis.
Risk of bias across studies	15^†^ Specify any assessment of risk of bias that may affect the cumulative evidence (e.g., publication bias, selective reporting within studies).
Additional analyses	16^†^ Describe methods of additional analyses (e.g., sensitivity or subgroup analyses, meta-regression), if done, indicating which were pre-specified.
Results	
Study selection	17^†^ Give numbers of studies screened, assessed for eligibility, and included in the review, with reasons for exclusions at each stage, ideally with a flow diagram.
Study characteristics	18* For each study, present characteristics that were extracted (e.g., study size, PICOS, follow-up period) and provide the citations of the included studies. Summarize details of the acupuncture intervention for each study in a table referring to TIDieR. 18a^‡^ Describe details of “De-qi” after acupuncture reported in the included studies.
Risk of bias within studies	19^†^ Present data on risk of bias of each study and, if available, any outcome-level assessment (see item 12).
Results of individual studies	20^†^ For all outcomes considered (benefits or harms), present, for each study: (a) simple summary data for each intervention group and (b) effect estimates and confidence intervals, ideally with a forest plot.
Synthesis of results	21^†^ Present results of each meta-analysis done, including confidence intervals and measures of consistency.
Risk of bias across studies	22^†^ Present results of any assessment of risk of bias across studies (see item 15).
Additional analysis	23^†^ Give results of additional analyses, if done (e.g., sensitivity or subgroup analyses, meta-regression [see item 16]).
Discussion	
Summary of evidence	24^†^ Summarize the main findings including the strength of evidence for each main outcome; consider their relevance to key groups (e.g., health care providers, users, and policy makers).
Limitations	25^†^ Discuss limitations at study and outcome level (e.g., risk of bias), and at review level (e.g., incomplete retrieval of identified research, reporting bias).
Conclusions	26^†^ Provide a general interpretation of the results in the context of other evidence, and implications for future research.
Funding	
Funding	27^†^ Describe sources of funding for the systematic review and other support (e.g., supply of data); role of funders for the systematic review.

Note: * modified original item ^†^ unmodified item. ^‡^ new extended item

**Title**

1* Identify the report as a systematic review, meta-analysis, or both; if applicable, state the specific type of acupuncture treatment, such as manual acupuncture or electroacupuncture.

The title should clearly reflect the objectives of the systematic review and ideally allow the readers and users to identify the population, intervention, comparison, outcome and study design [24]. If the study investigates the effect of a specific style of acupuncture on a particular condition, it should be stated in the title. If a systematic review is planned to examine a large category of interventions including acupuncture (e.g. non-pharmacological interventions [25]), it will be not necessary to mention acupuncture in the title

**Rationale**

3* Describe the rationale for what is already known about acupuncture for the target condition in the background; if applicable, state what is already known about the specific types of acupuncture to be studied, and describe whether there is any difference of the effects among different types of acupuncture.

As recommended by the *Cochrane Handbook* [26], the current state of the guideline’s application, significance, and the specific methods of acupuncture for the target disease, as well as the hypothesis and theoretical basis (e.g. the likely physiological mechanisms of acupuncture stimulation), should be clearly described in the Background/Introduction section. The authors could consider including a mini-review of existing systematic reviews on the chosen topic, summarizing the strengths and weaknesses of existing reviews and explain how the newly proposed review will address the weaknesses if applicable. If such reviews do not exist, this should be stated in the background section.

**Inclusion and exclusion criteria**

**6** ^**†**^ **Specify study characteristics (e.g., PICOS, length of follow-up) and report characteristics (e.g., years considered, language, publication status) used as criteria for eligibility, giving rationale.**

6a.1^**‡**^ **Describe the diagnostic criteria of the target condition in Western medicine.**

Diagnostic criteria are important to define a disease, and thus to clarify the population of interest. As there may be different diagnostic criteria for one condition, and the diagnostic criteria may change over time, this may result in different inclusion criteria of the participants. For example, a systematic review on acupuncture for hypertension used a blood pressure above 140/90 as inclusion criteria, which is the threshold for high blood pressure according to the editions of the American Heart Association guidelines published during the past 14 years [27]. But in 2017, AHA changed its threshold as 130/80 [28]. Reporting the diagnostic criteria with supporting literature being referred can help to clarify the scope of the systematic review.

6a.2^**‡**^ **If applicable, describe the diagnostic criteria in terms of traditional medicine, such as traditional Chinese medicine.**

Acupuncture is a type of traditional medicine, and therefore research in acupuncture sometimes uses diagnostic criteria and syndrome classification in terms of traditional medicine, which often differ from those in Western medicine. This information is however often omitted in the final report. Authors should report the diagnostic criteria according to traditional medicine when using these classifications to include patients.

6b^**‡**^ **Describe the types of acupuncture to be included, such as traditional acupuncture, electroacupuncture, or fire acupuncture.**

The types of acupuncture interventions are diverse, including traditional acupuncture (i.e. manual acupuncture with classical needle manipulation), electroacupuncture, ear acupuncture, scalp acupuncture, wrist-ankle acupuncture, and others [29]. The effect of different types of acupuncture can differ from each other [30, 31], and the effect of acupuncture interventions may change by stage of the disease [32]. Systematic reviewers should clarify which types of acupuncture will be included for analysis. In addition, the adjunctive therapies including moxibustion, cupping, herbal injections, heat lamps or guasha should also be clarified if used as eligible criteria.

6c^**‡**^ **If applicable, report measures for therapeutic effects using the terminology of either traditional medicine (e.g. syndrome score for syndrome remission) or Western medicine (e.g. scales for pain intensity).**

Measures for therapeutic effects in both traditional medicine [33] and Western medicine are important to understand how acupuncture works. If authors took such outcomes as eligibility criteria, they should describe the possible measurements of such outcomes in the inclusion and exclusion criteria, because many outcomes can be defined with varying measurements. For example, pain intensity has four commonly used measurement scales [34].

**Information sources**

7* Describe all sources of information (e.g., databases with dates of coverage, contact with study authors to identify additional studies) in the search, and report the date of the last search. If applicable, report the databases or complementary search methods for acupuncture or traditional medicine.

Many databases focusing specifically on acupuncture or traditional medicine have become increasingly mature, and they can provide a considerable amount of information for Traditional Chinese Medicine (TCM), Korean medicine and Japanese traditional medicine [35]. Additionally, in some non-English-speaking countries, especially in China, abundant acupuncture-related research continues to be published in local language, and this literature can usually be found in country-based databases only. It is therefore necessary to provide the source when used [36].

**Literature searches**

8* Present full electronic search strategy for at least one commonly used database (e.g. MEDLINE), including any limits used, such that it could be repeated. If applicable, include the full search strategy for at least a Western and a traditional medicine database for each systematic review where both were used.

The search strategy is an indispensable part of a systematic review. Currently, there are only few studies on how to search literature on acupuncture in specific TCM databases. The authors of the systematic reviews should develop search strategies that are rigorous and repeatable and provide a clear description for at least one commonly used database (like MEDLINE) and, when applicable, one acupuncture- or traditional medicine-tailored source, like the Allied and Complementary Medicine Database (AMED) and AcuTrials® (http://acutrials.ocom.edu).

**Data items**

11* List and define all variables for which data were sought (e.g., PICOS, funding sources) and any assumptions and simplifications made; describe data items about details of the acupuncture interventions and controls (e.g. sham acupuncture) referring to TIDieR when applicable.

In acupuncture, the effect is associated with several details of intervention, such as the type of needle and angle and depth of inserting, number of needles, duration of treatment, and acupoints, which vary between diseases [37, 38]. In addition, many researchers used sham acupuncture as a control to avoid nonspecific placebo effect and bias caused by the lack of blinding [39]and the design essentially depends on three factors: position, depth of the needle, and auxiliary tools [40–42]. The TIDieR checklist [43] provided a detailed guideline of intervention reporting, acupuncture systematic review authors could define the extraction items of acupuncture interventions referring to TIDieR.

**Study characteristics (one extended item)**

18* For each study, present characteristics that were extracted (e.g., study size, PICOS, follow-up period) and provide the citations of the included studies. Summarize details of the acupuncture intervention for each study in a table referring to TIDieR

This information corresponds to the item on data extraction, and should be presented with corresponding results referring to TIDieR items. If the information is insufficiently reported and cannot be obtained by contacting authors of the included studies, then authors should describe this information as “not reported” in their review.

**18a** ^**‡**^ **Describe details to refer to typical sensations associated with needling after acupuncture reported in the included studies.**

"De-qi" refers to the sensations typically associated with needling including soreness, numbness, heaviness, distension and aching at the insert position when the needle is inserted into acupoints of a certain depth during needling [44]. Seeking De-qi, as well as the time and strength of Qi feeling, affects the clinical efficacy of acupuncture [45, 46]. The current research on De-qi is getting increasingly mature with a growing number of studies being conducted. Systematic reviews should therefore extract and report this information together with how De-qi sensation was measured (e.g. specific scales) from the included studies if available, because there are different scales to quantify De-qi sensations, of which the rationales may not completely agree with each other [47]. Considering the controversies on the relation of De-qi and therapeutic effect, to provide De-qi details could provide future researchers with needed information to further exploration.

In addition to present the checklist, we also provided examples for each item from existing acupuncture systematic reviews and meta-analyses in Additional file 3.

## Discussion

With the aim to optimize reporting of systematic reviews focusing on acupuncture interventions for specific conditions, the PRISMA for Acupuncture checklist can be used for systematic reviews studying specific acupuncture, as well as a large category of interventions that include acupuncture, such as non-pharmacological interventions. The PRISMA for Acupuncture are developed for authors of systematic reviews on acupuncture, journal editors, peer reviewers and methodologists. Besides reporting, PRISMA for Acupuncture can also be used to evaluate the current condition of reporting, and to help journals identify acupuncture systematic reviews of higher quality. PRISMA for Acupuncture can also assist with the early design and development of protocols of acupuncture systematic reviews as other reporting guidelines do [48].

As with other extension of reporting guidelines, the PRISMA for Acupuncture checklist should be used together with the original PRISMA checklist, because some items in PRISMA are universally applicable for systematic reviews for different interventions. Referring to both, where relevant, and other PRISMA guidance (e.g. PRISMA for Protocols [9] can effectively ensure the rigor, transparency and integrity of reporting in acupuncture systematic reviews. To make it easier to use, we created an integrated checklist including both the new or modified items and the unchanged PRISMA items, so that users could refer to one combined checklist.

The implementation of PRISMA for Acupuncture requires continuous promotion of its use. We will continue to share the PRISMA for Acupuncture by presenting our results of international conferences and seminars, and contacting journals for endorsement. This reporting guideline will be published in both Chinese and English to promote its spread and increase its applicability, and additional translations are also encouraged as necessary, which will hopefully improve the accessibility. All the documents will be available on the PRISMA website (http://prisma-statement.org/), and the working group welcomes and collects comments and feedback from those in research or practice in order to revise PRISMA for Acupuncture and keep it up to date. We will also monitor the application and evaluate the effect of PRISMA for Acupuncture continuously, and when necessary, update it according to users’ feedback and the latest evidence.

## Conclusion

Ultimately, a checklist for the reporting of systematic review on acupuncture were formed. Although several extensions of PRISMA have been developed, covering aspects of study design, type of data, population, intervention, and outcome, PRISMA for Acupuncture supplements the relevant items for reporting issues specific for acupuncture interventions that were not considered in PRISMA and other extensions. Developed following recommendations for the development of reporting guidelines and integrated and extensive survey of evidence users, we believe the PRISMA for Acupuncture checklist will be a useful tool to promote the transparent reporting on acupuncture in systematic review, thus to achieve a better use in practice.

## Additional files

Additional file 1: Results of the Delphi process. (DOCX 27 kb)

Additional file 2: Results of the pilot test of the PRISMA for Acupuncture checklist. (DOCX 27 kb)

Additional file 3: Examples of reporting items in systematic reviews for Acupuncture. (DOCX 278 kb)

## Data Availability

All data generated or analysed during this study are included in this article and its supplementary files.

## Abbreviations

EQUATOR: Enhancing the QUAlity and Transparency Of health Research
PICOS: Population, Intervention, Outcome, Study design
PRISMA: Preferred Reporting Items for Systematic reviews and Meta-Analyses
RIGHT: Reporting Items for practice Guidelines in HealThcare
STRICTA: STandards for Reporting Interventions in Clinical Trials of Acupuncture
TCM: Traditional Chinese Medicine
TIDieR: Template for Intervention Description and Replication
