# Supplementary material for: Reporting items for systematic reviews and meta-analyses of acupuncture: the PRISMA for acupuncture checklist
Source: BMC Complement Altern Med. 2019 Aug 12;19:208. doi: 10.1186/s12906-019-2624-3 (PMC6689876; doi:10.1186/s12906-019-2624-3)
Supplement: Supplementary file 1 — Results of the Delphi process. (DOCX 27 kb) [file 12906_2019_2624_MOESM1_ESM.docx]

**Supplementary file 1: Results of the Delphi process**

**Table 1 The 1^st^ round**

| **Items** | **Score** | **Comments** |
| --- | --- | --- |
| **1. Title: Identify the report as a systematic reviews, meta-analysis, or both for acupuncture.** | 90.70%§ |  |
| **3. Background/Introduction: Provide the theoretical basis of acupuncture used for the target disease in** | 87.00%§ |  |
| **4. Objectives: Provide the style of acupuncture treatment (e.g. traditional Chinese acupuncture, South Korean acupuncture) in background/introduction** | 78.70% | Comment 1: Not sure about the differences.  Comment 2: It’s unnecessary to provide such detailed information in the objective, it could be presented in the methods. |
| **6a. Inclusion criteria: Provide the diagnostic criteria in methods (TCM syndrome and/or diagnostic criteria of diseases according to Western medicine)** | 91.70% | Comment 1: TCM syndrome is insignificant for diagnosis, we always use the Western medical diagnostic criteria.  Comment 2: TCM and Western medicine are two different systems, and we should not use them together. The TCM diagnostic criteria is enough.  Comment 4: They should be simultaneously employed.  Comment 5: It’s unnecessary to provide such detailed information, target population is enough. |
| **6b. Inclusion criteria: Basic information of participants, including demographic characteristics (age, gender, etc.), physical quality, and medical records in TCM and western medicine.** | 75.00% | Comment 1: Physical quality is inappropriate, it should be moved.  Comment 2: It should not be so cumbersome. |
| **6c. Provide types of acupuncture interventions in methods (e.g. type of acupunctures like percussopunctator and needles, and any other intervention like sham acupuncture)** | 94.40% | Comment 1: Sham acupuncture does not belong to acupuncture, the main purpose of acupuncture is for blinding.  Comment 2: Percussopunctator, filiform needle, and three-edged needle are different needles.  Comment 3: Could be combined with 6.4. |
| **6d. Provide details of acupuncture interventions in methods (e.g. number of needles, names of acupoint, depth of puncture, relevant body response, needling manipulation, time for needle retention, types of needles)** | 87.00% | Comment 1: The expression is different from the clinical practice, you should clearly state the number means how many or what the frequency.  Comment 2: You should include the information about dose (including the intensity and the frequency).  Comment 3: Maybe the original research have no relevant information, so it’s unnecessary.  Comment 4: These are the extended requirements, if too detailed, there may have no eligible studies. |
| **6e. Provide indicators of effect in methods (e.g. Visual Analogue Scale (VAS)** | 88.00% | Comment 1: The follow-up time should be presented separately.  Comment 2: The PRISMA statement have already required. |
| **18a. Provide the qualification (e.g. career and other experience) of acupuncture clinicians in methods** | 88.00% | Comment 1: Too many contents.  Comment 2: The confounding factors and personal qualifications should be separated. |
| **18b. Provide the follow-up time along with rationality in results** | 88.00% | Comment 1: The PRISMA statement have already required. |

§Included

**Table 2 The 2^nd^ round**

| **Items** | **Score** | **Comments** |
| --- | --- | --- |
| **6a.1 TCM syndrome differentiation for specific diseases** | 69.00%§ | Comment 1: The efficacy of acupuncture is based on changes in syndrome, and characteristics of syndrome are the clinical features of TCM and reflect the thinking of TCM syndrome differentiation. This information should be presented and could be related to the target intervention.  Comment 2: It should be tailored for the diagnosis criteria of a specific symptom of a disease.  Comment 3: Acupuncture can be done by both TCM practitioners and Western medicine practitioners depending on the purpose of the study, for example, acupuncture in neurology department could be done without syndrome differentiation. So the systematic review should be considered whether it is purely for TCM or pure Western medical systematic review, or both.  Comment 4: Most syndromes are subjective outcomes, and there is no clear and objective standard. Although it is very important in TCM, the lack of unified standards may confuse foreign researchers. So I recommended only to provide Western medicine diagnosis criteria.  Comment 7: TCM syndromes are mostly symptoms, which do not have a specific significance for diagnosis.  Comment 8: Syndrome type is important for recipe, but syndrome differentiation in acupuncture is mainly on meridians and collaterals.  Comment 10: Syndromes are always associated with drugs. |
| **6a.2 Provide western "disease" diagnostic criteria** | 88.80%§ | Comment 1: This item is necessary to present the diagnosis of modern medicine.  Comment 3: It is of great significance to promote the clinical application of acupuncture and enhance the understanding of acupuncture in Western medicine. It is conducive to internationalization of acupuncture research results.  Comment 4: If necessary, it should be noted that 6a-6c should not be the inclusion criteria for a systematic review. Just think that systematic review is a collection of multiple RCTs and each RCT may use different diagnostic criteria. How can they be listed together? Look at the original item, it also says "research features (PICOS and follow-up)","reporting characteristics (such as the search years, language and publication)", and does not refer to the diagnostic criteria. If the researcher is certain that the included study must contain this, it should be the supplement to the original item, not a replacement.  Comment 5: Ensure the identity of the study objects.  Comment 6: The correspondence of TCM symptoms and Western medicine diagnosis should be presented in the discussion. TCM and Western medicine should not be roughly put together, because they are two completely different system. Inferences can be made in the discussion. |
| **6b When applicable, expounded the subject of the syndrome or disease stage, history of Chinese and Western medicine treatment** | 67.20% | Comment 1: Explain it if necessary.  Comment 2: It is unnecessary to make such detailed description in the inclusion criteria.  Comment 3: It had little effect on acupuncture treatment.  Comment 4: Syndromes or disease staging of subjects should be described to ensure baseline homogeneity.  Comment 5: It is helpful for doctors to learn from or recommend to other patients if provided with treatment history?  Comment 6: Disease stage, course of disease and degree of severity are essential, whereas syndromes and treatment history are not recommended to be included.  Comment 7: A simple description of disease staging could be provided.  Comment 8: It may affect the intervention effect.  Comment 9: It is not easy to regulate because of the wide individual differences.  Comment 10: For some diseases, interventions for different stages are different, and the treatment records of included objects have certain impacts on the accuracy of results. However, the medical records and other information in the literature rarely mentioned in the clinical research, and the authenticity of document also needs to be further confirmed. So the importance level of this item is three, and I proposed to provide it as optional.  Comment 11: First treatment in disease spectrum of acupuncture is common.  This item is to further promote the homogeneity, but too many limitation is not conducive to the exploration. |
| **6c. Acupuncture intervention types, such as general acupuncture, electro-acupuncture, fire needle and so on** | 94.80%§ | Comment 1: To provide the type of acupuncture can provide reference for clinicians.  Comment 2: Accurate and detailed description of interventions is particularly important.  Comment 3: You should include TCM acupuncture, Kampo medicine acupuncture, Korean medicine acupuncture etc.  Comment 4: It directly affects the decision of the normal method and its efficacy.  Comment 5: It can be applied with less emphasis, and you should consider the potential basis of TCM theory if emphasizing it. |
| **6d.1. If applicable, provide indexes for therapeutic effects for syndrome remission in Chinese medicine, such as syndrome score.** | 81.00%§ | Comment 1: It is recommended as a necessary item.  Comment 2: It is important but not a mandatory option when considering the applicability of guideline.  Comment 3: Provide judgment criteria for outcome, because the same outcome may have different criteria. This is helpful for clinicians to understand.  Comment 4: If we only mentioned "symptom relief" here, it may mislead the readers that TCM can only relieve symptoms rather than cure disease.  Comment 5: Whether the evaluation criteria for efficacy is unified will have impact on assessment and heterogeneity.  Comment 6: If we use this as inclusion criteria, there maybe few eligible literatures.  Comment 7: Reporting objective outcome would be helpful for the quality assessment of the included studies and conversion and statistical analysis of data.  Comment 8: Making the symptom remission the primary outcome of TCM.  Comment 9: The main purpose of acupuncture therapy is to relieve symptoms, so symptom relief can be the outcome. A recognized TCM symptom scale but the self-produced scale is recommended to be an outcome.  Comment 10: Syndrome score is the diagnostic criteria but the standard for effect; symptom score is more significant as an outcome.  Comment 11: Symptom remission assessment is the key to TCM efficacy.  Comment 12: It is recommended not to use syndrome score indicator as an example.  Comment 13: Syndrome score is significant for TCM. |
| **6d.2. If applicable, provide indexes for therapeutic effects in western medicine, such as visual analogue scale.** | 93.10%§ | Comment 1: It is recommended as a necessary item.  Comment 2: Western evaluation criterion need to be presented.  Comment 3: Though important, TCM syndrome is mostly a subjective judgment without objective standard, and may make others hard to understand. So it is recommended only to provide Western medicine diagnosis.  Comment 4: It affects the efficacy evaluation, heterogeneity and method for meta analysis.  Comment 5: If this criterion is necessary, then too many literatures might be excluded.  Comment 7: At present, most acupuncture studies combine TCM syndrome scale with Western medical indicators. For a specific disease, widely recognized Western medicine indicators are important and essential.  Comment 8: Quantification of therapeutic effects is very important.  Comment 9: It directly relates to the accuracy and credibility of evaluation.  Comment 10: Western medical and TCM indicators can cover the shortages of each other.  Comment 11: "When applicable", this is more flexible.  Comment 12: It recommended to form a set of efficacy evaluation criteria regardless of TCM or Western medicine. It should be an interdisciplinary and unified standard without distinction of Chinese and Western medicine. |
| **7.Describe information sources, including databases or complement search for acupuncture or Chinese medicine.** | 86.20%§ | Comment 1: This is necessary, otherwise it could be easy to miss important literature.  Comment 2: It can provide reference for clinicians or for researchers to carry out systematic reviews.  Comment 4: It can be provided, but it is not critical.  Comment 5: It is important to judge whether the search is comprehensive or not.  Comment 6: The sources, including conference papers and other gray literature, relevant journals which need manual retrieval, can be included in the systematic review.  Comment 7: Many keywords of TCM literature are inappropriate, so some documents cannot be retrieved in several databases. |
| **8.Provide the search strategy to be used in databases or complement search for acupuncture or TCM.** | 88.80%§ | Comment 1: It is good for repeatability, also easy to judge whether the search is comprehensive and the search strategy is appropriate or not  Comment 2: Professional TCM database needs to be searched.  Comment 3: There is no evidence indicates that the search strategy for these databases is very different from other databases.  Comment 4: We should retrieve all the evidence as we can, but there are only few databases tailored for acupuncture or TCM, and they have not included all studies.  Comment 5: Describing simply and clearly is ok.  Comment 6: It may differ from the standard search strategy.  Comment 7: Special situations should be considered. |
| **11a. Describe details of acupuncture intervention, including the needle type, acupoints, inserting depth, retention time, frequency and courses).** | 93.10%§ | Comment 1: These factors are closely related to the effectiveness of acupuncture, and need to be reported in detail.  Comment 2: It should also include: the angle of inserting, stimulation (including electric stimulation, hand stimulation, etc.), needle type (including length, diameter etc.)  Comment 3: It is necessary.  Comment 4: The information about acupuncture treatment protocols in the included studies directly affect judgments of the efficacy.  Comment 5: It is directly related to efficacy and repeatability.  Comment 6: It overlaps the latter below, I recommended not to present here. |
| **11b. If applicable, describe implementation details of sham acupuncture intervention.** | 84.80%§ | Comment 1: These factors are closely related to the efficacy of acupuncture, and need to be reported with detail.  Comment 2: Some clinical trials may not use sham acupuncture as control group, so the proposed item should describe the collection of blank or sham acupuncture or other similar acupuncture stimuli.  Comment 3: Is the method in control group appropriate?  Comment 4: The current methods of sham acupuncture need to be studied.  Comment 5: It is important, but just for the interpretive studies. |
| **11c. Describe the information about “De-qi” after acupuncture.** | 75.00%§ | Comment 1: This information is very important for acupuncture, it can help clinicians implement acupuncture.  Comment 2: Because the description of Deqi has not been unified yet, detailed description is of little significance.  Comment 3: I suggest to describe it as organism response (including Deqi or muscle twitch reaction), "Deqi" is not accurate enough.  Comment 4: Deqi is the premise of success implementation of acupuncture.  Comment 5: It has something to do with efficacy, but foreign research may lack this information.  Comment 6: "Deqi" is actually the subjective judgment of the doctor on the success of acupuncture, which is theoretically important but the lack of objective criteria.  Comment 7: We should determine the clinically utility of the "Deqi".  Comment 9: According to the purpose of systematic review and specific diseases, choose whether to collect "Deqi" information.  Comment 10: In general, when the needle is inserted, it could be elicited.  Comment 11: "Deqi" is complex and is influenced by the practitioners’ skill and the condition of patients, so we should prudent whether it is ok or not. |
| **18a. Describe details of acupuncture intervention, including type of acupuncture, manipulation technique, needle type, acupoint, frequency, retention time, and courses. (see Item 6c, 11a)** | 94.00%§ | Comment 1: These factors are closely related to the efficacy of acupuncture, and need to be reported in detail.  It should also include: acupuncture type (Chinese medicine acupuncture, Kampo medicine acupuncture, Korean medicine acupuncture), angle of inserting, stimulation (including electric stimulation, hand stimulation, etc.), needle type (including length, diameter, etc.)  Comment 3: If a systematic review reported the detailed content, it may make a long body of literature. The the details of the intervention implementation and the extent of how detailed it should be all need evidence, if studies showed little impact of these information on the results, there is no need to report.  Comment 4: My opinion may not accurate because I’m not an expert in acupuncture. But I believe it is necessary to report it if sham acupuncture as a control intervention could facilitate the reduction of bias.  Comment 5: The details of acupuncture implementation are necessary, but which details should be described depend on the purpose and disease of systematic reviews.  Comment 6: It was associated with efficacy and repeatability. |
| **18b. If applicable, describe implementation details of fake acupuncture intervention.** | 97.30%§ | Comment 1: Detailed description of the control method is needed.  Comment 2: It could help determine the true effect of acupuncture.  Comment 3: As previous suggestion, it is recommended to describe it as blank controls, sham acupuncture or other similar stimulation controls.  Comment 4: Sham acupuncture is difficult to conduct, so we should specify the implementation methods that allow peers to understand it can achieve real comfort effect.  Comment 6: For there are currently various control interventions in acupuncture studies, the control group does not always use sham acupuncture, but results from sham acupuncture are more convincing. I recommended to keep this item.  Comment 7: It is rather important for the purpose of interpretive studies. |
| **18c. Describe the duration of acupuncture implementation and the follow-up period, for example the blood pressure at the end of treatment and six months after treatment finished.** | 95.70%§ | Comment 1: Necessary.  Comment 2: Some effect (like neuro-regulation) appears immediately, but some (like the immune regulation) often takes a long time be obtained.  Comment 3: The time point for measuring the results is relevant to the efficacy.  Comment 4: If follow-up implemented, long-term efficacy should be reported.  Comment 5: It is not a mandatory option, because there are limited acupuncture researches have the follow-up period currently. This option can be retained.  Comment 6: Follow-up efficacy may be one of the advantages of acupuncture. |
| **18d. Describe the background of participating acupuncturists (qualification or professional affiliation, years in acupuncture practice, other relevant experience)** | 69.80% | Comment 1: Simple description is OK.  Comment 2: This information is very important and can influence the effectiveness of acupuncture, meanwhile it helps determine the source of heterogeneity or bias.  Comment 3: Qualification is not equal to high level.  Comment 4: The focus of this issue is to describe the consistency among performers of acupuncture, not necessarily related to the qualification.  Comment 5: Acupuncture performers’ qualification, occupation time and other factors have certain impact on the efficacy of acupuncture therapy, but seldom literatures have reported this issue, especially the Chinese literatures. It is recommended as an option.  Comment 6: It is not required. Because basically, the consistency can be guaranteed after training.  Comment 7: This may closely relate to efficacy.  Comment 8: The background of acupuncture practitioners is complex, and the qualification approval of TCM practitioners is quite different from Western medicine.  Comment 9: Good point, but may be difficult to implement. |
|  |  | Comment 3: The systematic review should provide search strategies for at least one Chinese database and one non-Chinese database.  Response: the PRISMA statement has already explained. |

§Included

**Table 3 The 3^rd^ round**

| **Items** | **Score** | **Comments** |
| --- | --- | --- |
| **Provide the "syndrome-type" of TCM for specific diseases** | 69.00% | Comment 1: “syndrome-type” is the core of disease diagnosis in TCM  Comment 2: syndrome is the core and feature of clinical diagnosis in TCM, but the syndrome would be better reflected tailored for the characteristics of acupuncture, rather than indiscriminately using the syndrome from TCM internal medicine syndrome differentiation system.  Comment 3: It can reflect the feature of TCM, then determine whether the diagnosis of research objects is accurate or not, and whether it is related to the applicability of results.  Comment 4: syndrome type is one principle of TCM and part of the basis to select acupoints for prescription, it has some guiding effects for acupuncture.  Comment 5: Acupuncture treatment is not very related to syndrome type, but is very related to symptom. It is recommended to use "symptom" instead of "syndrome".  Comment 6: The participants are not treated according to the syndrome differentiation, it is not necessary to provide “syndrome-type”  Comment 7: Syndrome-type is ambiguous, which makes it difficult to determine the scope of the topic.  Comment 8: Acupuncture is also a kind of treatment which should be based on syndrome differentiation  Comment 9: Based on the research objectives, researchers need to state “syndrome-type”.  Comment 10: Researchers can provide TCM “syndrome-type”, but not necessarily as the inclusion criteria, because acupuncture sometimes is performed according to diseases and sometimes according to the syndrome-type, so add “syndrome-type” is optional.  Comment 11: I suggest to list this as supplement, because it can generalize TCM and enable the comparison between the TCM and western medicine.  Comment 12: Acupuncture is different from TCM, for which one disease only has one prescription, and add specific acupoints according to syndrome type is common.  Comment 13: The diagnostic criteria of TCM is special, and need to be reported clearly.  Comment 14: Syndrome-type can reflect the characteristic of TCM, whether to add TCM syndrome is closely related to the diseases. TCM has been used more in internal medicine, and there are more syndrome in internal medicine, where the prescription needs to be based on syndrome differentiation. The acupuncture is mostly used in neuromuscular diseases, for which the acupoint selection is mostly based on channels identification, whereas the syndrome differentiation is always used in internal medicine.  Comment 15: The key point of acupuncture is the reinforcing and reducing methods, and the De-qi, too detailed “syndrome-type” is unnecessary. |
| **If applicable, describe syndrome or disease stage and medical record in TCM and Western medicine.** | 75.90%§ | Comment 1: Syndrome-type was mentioned before, and this one can be merged with the previous item.  Comment 2: Syndrome and disease stages are both necessary  Comment 3: Disease stages would have effect on efficacy of the intervention, we need to consider it.  Comment 4: Syndrome has been mentioned in item 6a, it is enough to only mention disease stage here, and the syndrome should be optional.  Comment 6: Acupuncture is different from TCM, for which one disease only has one prescription, and add specific acupoints according to syndrome type is common.  Comment 7: Clear description of disease characteristics can more specifically use of results.  Comment 8: If added, add the syndrome of participants  Comment 9: It is important to describe the stage, because it is obviously related to the effect.  Comment 10: It can be more accurate to allocate treatment based on syndrome differentiation.  Comment 11: Stage may not be restricted in inclusion criteria, and the following subgroup analysis can reflect different stage.  Comment 12: If the study is closely related to the syndrome or disease stage, it can be considered  Comment 13: It is needed in some cases. |
| **Description of personal qualifications for the acupuncture performer, including qualification to obtain employment (such as accept training, get qualification certificates), employment time and other related experience, etc.** | 68.10% | Comment 1: Acupuncturists, as part of the heterogeneity source, are likely to influence the effect, so researchers need clear and detailed reporting.  Comment 2: Current English papers often require a simple description of acupuncturists’ qualification, which aims to control the bias arising from individual factors. It is recommended to report this information.  Comment 3: These are related to the ability of acupuncturists  Comment 4: Personal qualification should be managed by medical management department. If a person is not qualified, he/ she should not be a practitioner.  Comment 5: It seems important, but may not be available in most of the literatures.  Comment 6: Acupuncture should be implemented by a qualified individual, but not necessarily report the personal qualifications.  Comment 7: There are more and more clinical reports describe the personnel qualifications, but most of the literatures did not mentioned this information. I don’t recommend to add this item because this may increase the sub-group numbers of meta-analysis.  Comment 8: As the society progressed, full-time undergraduate is the minimum requirement of being an acupuncturist, so it should be standardized.  Comment 9: Whether having the qualification and the depth of qualification are the governments’ and associations’ responsibility. If there is no qualification evaluation system, whether trained or not and how long a practitioner worked are not reliable standards.  Comment 10: The key is: whether it is the qualification or employment time.  Comment 11: For acupuncture with more complex operation, it needs to be clear.  Comment 12: Can be used as a supplementary to provide a reference for judging the generalizability of the study.  Comment 13: Description of the qualification is mainly about the professional title. For example: residents, attending physician, associate senior doctor, chief physician.  Comment 14: Being trained or not directly affects the effect of acupuncture. |

§Included
