# Supplementary material for: Reporting items for systematic reviews and meta-analyses of acupuncture: the PRISMA for acupuncture checklist
Source: BMC Complement Altern Med. 2019 Aug 12;19:208. doi: 10.1186/s12906-019-2624-3 (PMC6689876; doi:10.1186/s12906-019-2624-3)
Supplement: Supplementary file 2 — Results of the pilot test of the PRISMA for Acupuncture checklist. (DOCX 27 kb) [file 12906_2019_2624_MOESM2_ESM.docx]

**Supplementary file 2: Results of the pilot test of the PRISMA for Acupuncture checklist.**

| **Subjects** | **PRISMA for Acupuncture checklist** | |
| --- | --- | --- |
| ***Title*** |  |  |
| **Title** | 1^*^ Identify the report as a systematic review, meta-analysis, or both; if applicable, state the specific type of acupuncture treatment, such as manual acupuncture or electroacupuncture. | |
| ***Summary of the pilot testing*** | - *Characteristics of the acupuncture systematic reviews:*   All 27 SRs reported intervention as acupuncture; ten (37%) reported using a specific type of acupuncture (e.g. electro- acupuncture).   - *Comments from the authors of acupuncture SRs:*   A: Suggest using PICOS format for the title when possible.  **Response**: agree, and this was already stated in the original PRISMA statement. | |
| ***Abstract*** |  |  |
| **Structured summary** | 2^†^ Provide a structured summary including, as applicable: background; objectives; data sources; study eligibility criteria, participants, and interventions; study appraisal and synthesis methods; results; limitations; conclusions and implications of key findings; systematic review registration number. | |
| ***Introduction*** |  |  |
| **Rationale** | 3^*^ Describe the rationale on what is already known about acupuncture for the target condition in the background; if applicable, state if there is any difference of effects among different types of acupuncture. | |
| ***Summary of the pilot testing*** | - *Characteristics of the acupuncture systematic reviews:*   Three (11%) SRs described the difference of the effects among different types of acupuncture. All of them focused on electro-acupuncture. Six (22%) SRs reported no information on the rationale for using acupuncture for the target condition, and two (7%) only simply mentioned the use of acupuncture for the condition without describing any rationale.   - *Comments from the authors of acupuncture SRs:*   A: Suggest to include a mini-review of existing systematic reviews, Cochrane reviews and meta-analyses on the chosen topic. If such reviews do not exist, it should state so in the background section. The mini-review should state the strengths and weaknesses of the existing reviews and how the newly proposed review will address these weaknesses if applicable.  **Response**: integrated as explanation in the full manuscript  C: suggest to describe as “*Describe the rationale on what is already known about acupuncture for the target condition in the background; if applicable, state the TCM theory underlying the treatment effect of the acupuncture for the target condition; state if there is any difference of effects among different types of acupuncture.*”  **Response**: integrated together with comment from author **I**, and the revised item is “*Describe the rationale on what is already known about acupuncture for the target condition in the background;* ***if applicable, state what is already known about the specific types of acupuncture to be studied,*** *and describe if there is any difference of the effects among different types of acupuncture.*”  F: The main conclusion for PRISMA must be that the rationale for the study is watertight: there must be a compelling reason to do the systematic review based on widespread use for the indication in question, lack of prior data (a key issue for reviews on pain conditions) and a reason to believe that there exist high quality trials that require review and collation.  **Response**: see *response* to author **A**. This comment was also forwarded to the PRISMA working group.  G: Unclear what does “in the background” mean?  I: Partly agree. Detailed description of the type of acupuncture researched is more necessary.  **Response**: see *response* to author **C** | |
| **Objectives** | 4^†^ Provide an explicit statement of questions being addressed with reference to participants, interventions, comparisons, outcomes, and study design (PICOS) | |
| ***Methods*** |  |  |
| **Protocol and registration** | 5^†^ Indicate if a review protocol exists, if and where it can be accessed (e.g., web address), and, if available, provide registration information including registration number. | |
| **Eligibility criteria** | 6^†^ Specify study characteristics (e.g., PICOS, length of follow-up) and report characteristics (e.g., years considered, language, publication status) used as criteria for eligibility, giving rationale.  6a.1^‡^ Describe the diagnostic criteria of the target condition in Western medicine.  6a.2^‡^ If applicable, describe the diagnostic criteria in Traditional Medicine, such as Traditional Chinese Medicine.  6b^‡^ Describe the types of acupuncture to be included, such as traditional acupuncture, electroacupuncture, or fire acupuncture.  6c^‡^ If applicable, report measures for therapeutic effects in both Traditional Medicine (e.g. syndrome score for syndrome remission and in Western medicine (e.g. pain intensity). | |
|  |  |  |
|  |  |  |
|  |  |  |
|  |  |  |
| ***Summary of the pilot testing*** | - *Characteristics of the acupuncture systematic reviews:*   6a.1-2^‡^ Six (22%) SRs and three (11%) SRs described the diagnostic criteria of the target condition in Western medicine and in Traditional Medicine respectively.  6b^‡^ Twelve (44%) SRs reported the types of acupuncture to be included in the eligibility criteria  6c^‡^ Thirteen (48%) SRs reported measures for therapeutic effects in terms of either Western Medicine or Traditional Medicine, such as the UPDRS score, standardized pain scores, or Traditional Chinese Medicine symptom score.   - *Comments from the authors of acupuncture SRs:*   A: Supporting literature, with preference on clinical guidelines, textbooks, existing Cochrane reviews or high quality RCTs, must be cited when selecting of outcome measures. Minimum clinical important difference should be stated if available.  **Response**: integrated as explanation in the full manuscript  B: Describe the duration and frequency of acupuncture  **Response**: this information was described in detail in data items.  C: suggest to describe as “*If applicable, report measures for therapeutic effects in Traditional Medicine (e.g. syndrome score for syndrome remission) or in Western medicine*”  **Response**: integrated in the revised item as “*If applicable, report measures for therapeutic effects using either Traditional Medicine (e.g. syndrome score for syndrome remission) or Western medicine (e.g. pain intensity) terminology.”*  D: Adjunctive therapies such as moxibustion, cupping, herbal injections into acupuncture points and dry needling, should be mentioned as well. Control treatments should also be mentioned. If the control is not active (i.e. waiting list only), it should be reported if there is any justifications for this. The control should not involve verum acupuncture, even if the needles are just placed without any manipulation.  **Response**: integrated as explanation in the full manuscript  G: Add: if there are exclusions by language, year, publication status, age, sex, justify those exclusions  K: “*If applicable, describe the diagnostic criteria in Traditional Medicine, such as Traditional Chinese Medicine.”* This can be confusing because Traditional Chinese Medicine could not be the example of diagnostic criteria in Traditional Medicine.  **Response**: agree. We adjusted the item as “*If applicable, describe the diagnostic criteria* ***in terms of*** *Traditional Medicine, such as Traditional Chinese Medicine.*” and added explanation in the full manuscript | |
| **Information sources** | 7^*^ Describe all information sources (e.g., databases with dates of coverage, contact with study authors to identify additional studies) in the search and date last searched. If applicable, report the databases or complementary search methods for Acupuncture or Traditional Medicine. | |
| ***Summary of the pilot testing*** | - *Characteristics of the acupuncture systematic reviews:*   Twenty-six (96%) SRs reported all data sources, and 22 contained both general databases and databases focusing on traditional medicine-based (e.g. AMED) or databases in languages other than English (e.g. China National Knowledge Infrastructure (CNKI), Chinese Biological Medical Database etc.).   - *Comments from the authors of acupuncture SRs:*   A: Given the relatively large volume of acupuncture literature that exists in Chinese databases, it is recommended to include at least one major Chinese database in the search, e.g. CNKI.  **Response**: integrated as explanation in the full manuscript | |
| **Search** | 8^*^ Present full electronic search strategy for at least one commonly used database (e.g. MEDLINE), including any limits used, such that it could be repeated; if applicable, include the full search strategy for at least one source (database or manual search for literatures) tailored for Acupuncture or Traditional Medicine. | |
| ***Summary of the pilot testing*** | - *Characteristics of the acupuncture systematic reviews:*   Full search strategy of at least one general database and one traditional medicine based database was provided in only one (4%) SR. Fifteen (55%) SRs provided only the terms in English and Chinese, or described the general search strategy for both English- and Chinese-language databases, without reporting database-tailored search strategies that could be repeated.   - *Comments from the authors of acupuncture SRs:*   H: I think that it is not necessary to provide the full search strategy for at least one source (database or manual search for literatures) tailored for Acupuncture or Traditional Medicine.  **Response**: for the potential big difference of search strategy between general database and Acupuncture or Traditional Medicine tailored databases, we kept the content unchanged.  J: Suggest change “MEDLINE” to “PubMed”.  **Response**: MEDLINE is a specific database while PubMed is platform including different databases, and we kept “MEDLINE” keep consistent with PRISMA statement. | |
| **Study selection** | 9^†^ State the process for selecting studies (i.e., screening, eligibility, included in systematic review, and, if applicable, included in the meta-analysis). | |
| **Data collection**  **process** | 10^†^ Describe method of data extraction from reports (e.g., piloted forms, independently, in duplicate) and any processes for obtaining and confirming data from investigators. | |
| **Data items** | 11^*^ List and define all variables for which data were sought (e.g., PICOS, funding sources) and any assumptions and simplifications made; describe data items about details of acupuncture interventions and controls (**e.g.,** sham acupuncture) referring to TIDieR when applicable.  11a^‡^ Describe data item about details of “De-qi” after acupuncture if applicable. | |
| ***Summary of the pilot testing*** | - *Characteristics of the acupuncture systematic reviews:*   Nineteen (73%) SRs listed and defined variables including information about intervention, while only eight (30%) mentioned the details of intervention to be extracted, including the type of needling intervention, technique, acupuncture points and number, frequency, retention time, treatment time, and accompanying interventions. Three (11%) SRs reported to have designed the extraction form based on the Standards for Reporting Interventions in Clinical Trials of Acupuncture (STRICTA) guidelines.  11a^‡^. No SR reported De-qi item in their data abstraction method, but five (19%) SRs abstracted the data on De-qi and reported this in their table of included study characteristics.   - *Comments from the authors of acupuncture SRs:*   D: I do not think “De-qi” should be a reportable item, as certain styles of acupuncture are not as focused on “De-qi”. It is also not applicable to electroacupuncture and laser acupuncture and many studies do not mention how “De-qi” was elicited and for how long, even if it was considered clinically important.  **Response**: considered together with the result from review of acupuncture systematic reviews, we moved this to the **Study Characteristics** under **Result** section  E: De-qi is not always available in the relevant acupuncture studies and the definition is different in different studies, thus I think this item is not necessary  **Response**: see response to author D  G: I doubt whether all of TIDieR is relevant. Provide numbers of relevant components. Or better yet: put entire sections of TIDieR that are relevant right into #11 and #18  **Response**: agree, and we provided example in Supplementary file 3, and we also used “when applicable” for reasonability.  I: Partly agree. It is hard to describe the “De-qi” in systematic review. If the study is an original research, 11a is necessary.  **Response**: see response to author D | |
| **Risk of bias in**  **individual studies** | 12^†^ Describe methods used for assessing risk of bias of individual studies (including specification of whether this was done at the study or outcome level), and how this information is to be used in any data synthesis. | |
| **Summary measures** | 13^†^ State the principal summary measures (e.g., risk ratio, difference in means). | |
| **Synthesis of results** | 14^†^ Describe the methods of handling data and combining results of studies, if done, including measures of consistency (e.g., I^2^) for each meta-analysis. | |
| **Risk of bias across**  **studies** | 15^†^ Specify any assessment of risk of bias that may affect the cumulative evidence (e.g., publication bias, selective reporting within studies). | |
| **Additional analyses** | 16^†^ Describe methods of additional analyses (e.g., sensitivity or subgroup analyses, meta-regression), if done, indicating which were pre-specified. | |
| ***Results*** |  |  |
| **Study selection** | 17^†^ Give numbers of studies screened, assessed for eligibility, and included in the review, with reasons for exclusions at each stage, ideally with a flow diagram. | |
| **Study characteristics** | 18^*^ For each study, present characteristics for which data were extracted (e.g., study size, PICOS, follow-up period) and provide the citations. Summarise details of the acupuncture intervention for each study in a table referring to TIDieR. | |
| ***Summary of the pilot testing*** | - *Characteristics of the acupuncture systematic reviews:*   There were 25 (93%) SRs reporting the intervention details in their table on summary of findings, including the following components: 1) specific acupuncture intervention, 2) location and number of acupoints, 3) needle type (diameter/length), 4) depth of insertion, 5) response sought (Deqi), 6) retention time, 7) frequency, 8) duration of one session and number of sessions, 10) treatment course, 11) other combined treatment and details, 12) electro-equipment, 13) practitioner’s qualification, 14) duration of follow-up. The completeness of details of interventions varied across SRs, with some mentioning only the technique or acupoints, and others covering all 17 subitems according to the STRICTA checklist. No SR mentioned the TIDieR checklist.   - *Comments from the authors of acupuncture SRs:*   J: In e.g., add “side effect” or “complication”.  **Response**: agree, and the “side effect” or “complication” could be covered by outcomes in PICOS | |
| **Risk of bias within**  **studies** | 19^†^ Present data on risk of bias of each study and, if available, any outcome-level assessment(see item 12). | |
| **Results of individual**  **studies** | 20^†^ For all outcomes considered (benefits or harms), present, for each study: (a) simple summary data for each intervention group and (b) effect estimates and confidence intervals, ideally with a forest plot. | |
| **Synthesis of results** | 21^†^ Present results of each meta-analysis done, including confidence intervals and measures of consistency. | |
| **Risk of bias across**  **studies** | 22^†^ Present results of any assessment of risk of bias across studies (see item 15). | |
| **Additional analysis** | 23^†^ Give results of additional analyses, if done (e.g., sensitivity or subgroup analyses, meta-regression [see item 16]). | |
| ***Discussion*** |  |  |
| **Summary of evidence** | 24^†^ Summarize the main findings including the strength of evidence for each main outcome; consider their relevance to key groups (e.g., health care providers, users, and policy  makers). | |
| **Limitations** | 25^†^ Discuss limitations at study and outcome level (e.g., risk of bias), and at review level (e.g., incomplete retrieval of identified research, reporting bias). | |
| **Conclusions** | 26^†^ Provide a general interpretation of the results in the context of other evidence, and implications for future research. | |
| ***Funding*** |  |  |
| **Funding** | 27^†^ Describe sources of funding for the systematic review and other support (e.g., supply of data); role of funders for the systematic review. | |

Notes:

* modified original item, † unmodified item, ‡ new extended item;

The capital characters A-K represent different authors of acupuncture systematic reviews. Two authors replied with no comment.
