# Supplementary material for: Reporting items for systematic reviews and meta-analyses of acupuncture: the PRISMA for acupuncture checklist
Source: BMC Complement Altern Med. 2019 Aug 12;19:208. doi: 10.1186/s12906-019-2624-3 (PMC6689876; doi:10.1186/s12906-019-2624-3)
Supplement: Supplementary file 3 — Examples of reporting items in systematic reviews for Acupuncture. (DOCX 278 kb) [file 12906_2019_2624_MOESM3_ESM.docx]

**Supplementary file 3:** **Examples of reporting items in systematic reviews for Acupuncture**

| **Subjects** | **Included items** |
| --- | --- |
| ***Title*** |  |
| **Title** | **1* Identify the report as a systematic review, meta-analysis, or both; if applicable, state the specific type of acupuncture treatment, such as manual acupuncture or electroacupuncture.**  *Example 1*: The Efficacy of Acupuncture for the Treatment of Sciatica: A Systematic Review and Meta-Analysis(1).  *Example 2*: Electroacupuncture for Post stroke Spasticity: A Systematic Review and Meta-Analysis(2). |
| ***Introduction*** |  |
| **Rationale** | **3* Describe the rationale on what is already known about acupuncture for the target condition in the background; if applicable, state what is already known about the specific types of acupuncture to be studied, and describe if there is any difference of the effects among different types of acupuncture.**  *Example 1*: Acupuncture involves complex theories of regulation of the five elements (fire, earth, metal, water, and wood), yin and yang, Qi, and blood and body fluids. By stimulating various meridian points disharmony and dysregulation of organ systems is corrected to relieve symptoms and restore natural internal homeostasis (Maciocia1989). Many studies in animals and humans have demonstrated that acupuncture can cause multiple biological responses (Wang 2001). These responses can occur both locally or close to the site of application (Jansen 1989) and at a distance, mediated mainly by the sensory neurons to many structures that are within the central nervous system (Magnusson 1994). The result is activation of pathways affecting various physiological systems in the brain as well as in the periphery(3).  *Example 2*: Acupuncture has been practised in China for a long time and increasingly popular in western countries in recent decades. When electrical stimulation is applied through a small electric current passing between pairs of acupuncture needles, it is then known as electro-acupuncture (EA). Compared to manual acupuncture, EA can provide a constant stimulation, whose intensity, frequency and duration are quantifiable. Therefore, on one hand, EA enables clinicians to apply a standardized treatment in clinical practice. On the other hand, when intervention could be specifically defined, studies on different parameters would be possible, and a relatively higher reporting quality may be achieved. A cross-over clinical trial had suggested that EA combined with traditional rehabilitation therapies could reduce spasticity of the wrist joint in chronic stroke survivors(2). |
| ***Methods*** |  |
| **Eligibility criteria** | **6† Specify study characteristics (e.g., PICOS, length of follow-up) and report characteristics (e.g., years considered, language, publication status) used as criteria for eligibility, giving rationale.**  **6a.1‡ Describe the diagnostic criteria of the target condition in Western medicine.**  *Example*: The diagnosis of RA was defined according to the criteria of the American Rheumatism Association(4). |
|  | **6a.2‡ If applicable, describe the diagnostic criteria in terms of Traditional Medicine, such as Traditional Chinese Medicine.**  *Example*: Participants: patients diagnosed with acne, regardless of their gender, age, course of disease, and source of cases. We referred to the following diagnostic criteria: "Guiding Principles for Clinical Study of New Chinese Medicines", “Criteria of diagnosis and therapeutic effect of diseases and syndromes in Chinese medicine”(5). |
|  | **6b‡ Describe the types of acupuncture to be included, such as traditional acupuncture, electroacupuncture, or fire acupuncture.**  *Example*: We included both traditional acupuncture, in which the needles are inserted in classical meridian points, and contemporary acupuncture, in which the needles are inserted in non-meridian or trigger points, regardless of the source of stimulation (for example, ear acupuncture, scalp acupuncture, abdominal acupuncture, wrist-ankle needle, fire needle, moxibustion with warming needle, or electrical stimulation, etc)(6). |
|  | **6c‡ If applicable, report measures for therapeutic effects using the terminology of either traditional medicine (e.g. syndrome score for syndrome remission) or Western medicine (e.g. pain intensity).**  *Example 1*: Inclusion criteria: … Clinical efficacy (cured, markedly effective, and effective) and invalid evaluation were used as the end-point, and the criteria for invalid evaluation was the syndrome score was reduced by less than 30% or 1/3(7).  *Example 2*: RCTs were included that used at least one of the four outcome measures considered to be important in the field of low-back pain: pain intensity (e.g., visual analog scale (VAS)), a global measure (e.g., overall improvement, proportion of patients recovered, subjective improvement of symptoms), back specific functional status (e.g., Roland Disability Scale, Oswestry Scale) and return to work (e.g., return to work status, number of days off work(8). |
| **Information sources** | **7* Describe all sources of information (e.g., databases with dates of coverage, contact with study authors to identify additional studies) in the search, and report the date of the last search. If applicable, report the databases or complementary search methods for acupuncture or traditional medicine.**  *Example1*: The following 16 databases will be searched from their inception to 14 May 2017: MEDLINE, the Cochrane Central Register of Controlled Trials, EMBASE, the Cumulative Index to Nursing and Allied Health Literature, the Allied and Complementary Medicine Database, three Chinese database (China National Knowledge Infrastructure, the Chongqing VIP Chinese Science and Technology Periodical Database and the Wanfang database) and eight Korean databases (Korean Medical Database, Korean Association of Medical Journal Editors, Korean Studies Information Service System, Korean National Assembly Digital Library, National Digital Science Library, Oriental Medicine Advanced Searching Integrated System, 'Database Periodical Information Academic and Korean Traditional Knowledge Portal'). The WHO International Clinical Trials Registry Platform will also be searched to retrieve the recently completed studies(9).  *Example2:* Searching other resources  In the meantime, we will also search medical journals in university libraries related to this topic, such as Journal of Clinical Acupuncture and Moxibustion (1985–2018.1), Chinese Acupuncture, and Moxibustion (1981-2018.1)(10). |
| **Literature Searches** | **8* Present full electronic search strategy for at least one commonly used database (e.g. MEDLINE), including any limits used, such that it could be repeated. If applicable, include the full search strategy for at least a Western and a traditional medicine database for each systematic review where both were used.**  *Example:*  Appendix 3. AMED search strategy(11)  AMED (SilverPlatter)  #1 explode ’CEREBROVASCULAR-DISORDERS’ in SH; #2 stroke* or poststroke* or cva*; #3 cerebrovascular or cerebral vascular; #4 cerebral or cerebellar or brainstem or vertebrobasilar; #5 infarct* or isch?emi* or thrombo* or apoplexy or emboli*; #6 #4 and #5; #7 cerebral or intracerebral or intracranial or parenchymal or brain or intraventricular or brainstem or cerebellar or infratentorial or supratentorial; #8 haemorrhage or haemorrhage or hematoma or hematoma; #9 #7 and #8; #10 #1 or #2 or #3 or #6 or #9; #11 explode ’ACUPUNCTURE-’ in SH; #12 acupunctur* or electro-acupuncture or electroacupuncture or meridians or acupoint* or needling or trigger point*; #13 #11 or #12; #14 #10 and #13 |
| **Data items** | **11* List and define all variables for which data were sought (e.g., PICOS, funding sources) and any assumptions and simplifications made; describe data items about details of the acupuncture interventions and controls (e.g.,** **sham acupuncture) referring to TIDieR when applicable.**  *Example*: We recorded information on details of authors, participants, study design, characteristics of intervention (acupuncture style, type of needle, number of needles, needling technique, needle sensation and number of treatments) and comparator, any adverse effects and baseline/end of study outcomes(12).  *Example*: In particular, we extracted exact diagnoses; headache classifications used; number and type of centers; age; sex; duration of disease; number of patients randomized, treated and analyzed; number of, and reasons for dropouts; duration of baseline, treatment and follow-up periods; details of acupuncture treatments (such as selection of points; number, frequency and duration of sessions; achievement of de-chi (an irradiating feeling considered to indicate effective needling); number, training and experience of acupuncturists)(13). |
| ***Results*** | |
| **Study characteristics** | **18* For each study, present characteristics that were extracted (e.g., study size, PICOS, follow-up period) and provide the citations of the included studies. Summarize details of the acupuncture intervention for each study in a table referring to TIDieR.**  **18a‡ Describe details to refer to typical sensations associated with needling after acupuncture reported in the included studies.**  *Example*(14):  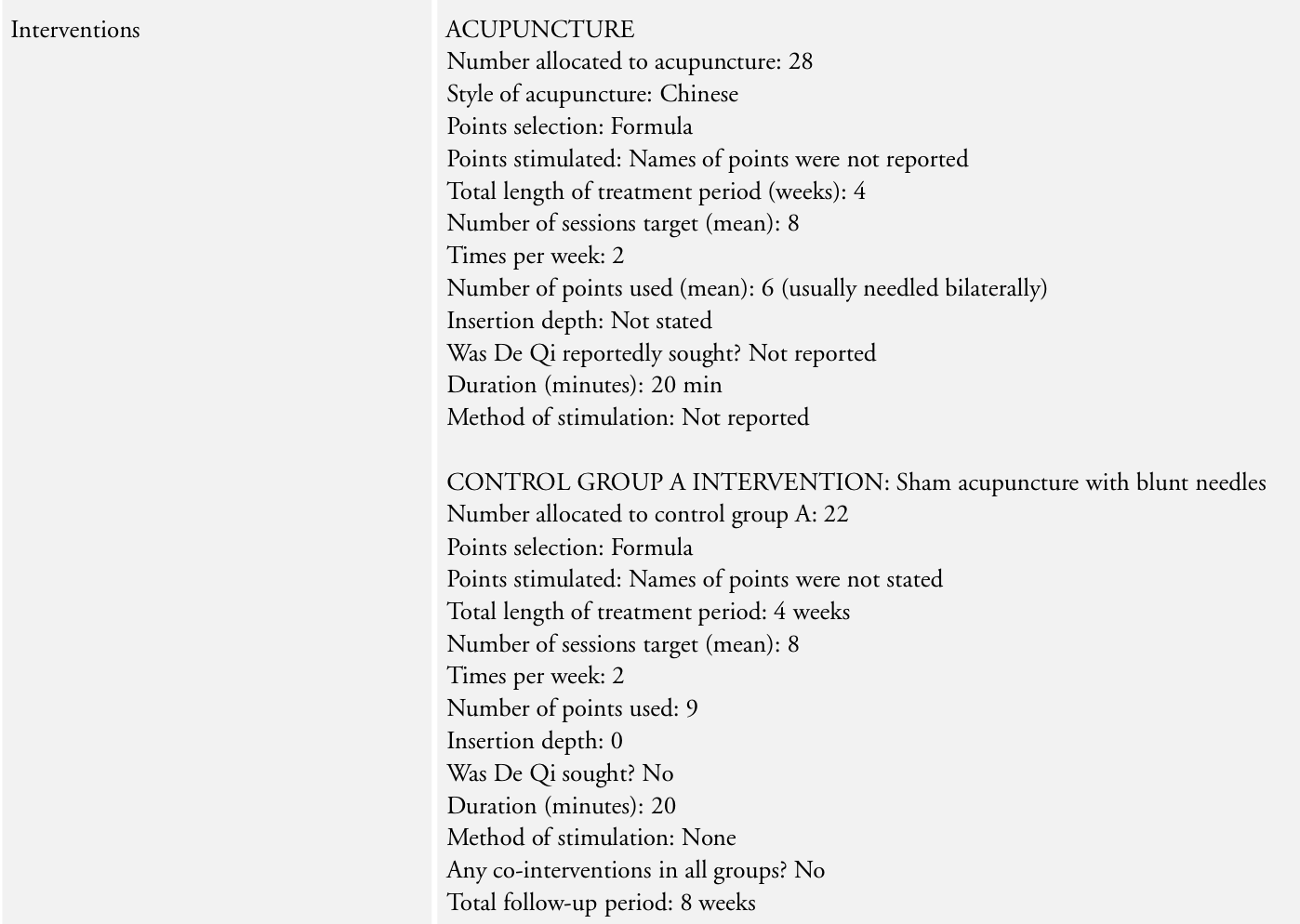 |
| * modified original item; † unmodified item; ‡ new extended item  **References of the examples:**  Ji M, Wang X, Chen M, Shen Y, Zhang X, Yang J. The efficacy of acupuncture for the treatment of sciatica: a systematic review and meta-analysis. Evid Based Complement Alternat Med, 2015, 2015 :192808.  Cai Y, Zhang CS, Liu S, Wen Z, Zhang AL, Guo X, et al. Electroacupuncture for Poststroke Spasticity: A Systematic Review and Meta-Analysis. Arch Phys Med Rehabil. 2017; 98(12):2578-2589.e4.  Cheuk DK, Wong V. Acupuncture for epilepsy. The Cochrane Library, 2006.  Brosseau L, Yonge KA, Welch V, Robinson VA, Tugwell P, Wells G, et al. Transcutaneous electrical nerve stimulation (TENS) for the treatment of rheumatoid arthritis in the hand. The Cochrane Library, 2003.  Qiuhong M, Lichang L, Jianqiong L, Xiuhong X, Shengxu W. Acupuncture and and moxibustion for cane: systematic review of randomized controlled trial. Guiding Journal of Traditional Chinese Medicine, 2015,21(12):76-83. [Article in Chinese]  Tulder MW, Cherkin DC, Berman B. Acupuncture for low back pain. The Cochrane Library, 1999 (2): CD001351-CD001351.  Wang T, Xu C, Pan K, Xiong H. Acupuncture and moxibustion for chronic fatigue syndrome in traditional Chinese medicine: a systematic review and meta-analysis. BMC Complement Altern Med. 2017;17(1):163.  Furlan AD, Van Tulder MW, Cherkin D, [Tsukayama H](https://www.ncbi.nlm.nih.gov/pubmed/?term=Tsukayama%20H%5BAuthor%5D&cauthor=true&cauthor_uid=15834340), [Lao L](https://www.ncbi.nlm.nih.gov/pubmed/?term=Lao%20L%5BAuthor%5D&cauthor=true&cauthor_uid=15834340), [Koes B](https://www.ncbi.nlm.nih.gov/pubmed/?term=Koes%20B%5BAuthor%5D&cauthor=true&cauthor_uid=15834340), et al. Acupuncture and dry‐needling for low back pain. The Cochrane Library, 2005.  Cho Y, Lee S, Kim J, Kang JW, Lee JD. Thread embedding acupuncture for musculoskeletal pain: a systematic review and meta-analysis protocol. BMJ Open. 2018 26;8(1): e015461.  Xiong J, Li H, Li X, Wang L, Zhao P, Meng D, et al. Electroacupuncture for postoperative pain management after total knee arthroplasty: Protocol for a systematic review and meta-analysis. Medicine (Baltimore). 2018 Mar;97(9): e0014.  Zhang SH, Liu M, Asplund K, Li L. Acupuncture for acute stroke. Stroke, 2005, 36(10): 2327-2328.  Paley CA, Johnson MI, Tashani OA, Bagnall AM. Acupuncture for cancer pain in adults. Cochrane Database Syst Rev. 2015 Oct 15;(10):CD007753.  Linde K, Allais G, Brinkhaus B, Manheimer E, Vickers A, White AR. Acupuncture for the prevention of tension‐type headache. The Cochrane Library, 2016.  Manheimer E, Cheng K, Wieland LS, Min LS, Shen X, Berman BM, et al. Acupuncture for treatment of irritable bowel syndrome. Cochrane Database Syst Rev. 2012 May 16;(5):CD005111.  Hurlow A, Bennett M I, Robb K A, et al. Transcutaneous electric nerve stimulation (TENS) for cancer pain in adults[J]. The Cochrane Library, 2012.  Cheong YC, Hung Yu Ng E, Ledger WL Acupuncture and assisted conception (Review)  Linde K, Allais G, Brinkhaus B, et al. Acupuncture for migraine prophylaxis (Review) | |
